# Novel pharmacist-led intervention secures the minimally important difference (MID) in Asthma Control Test (ACT) score: better outcomes for patients and the healthcare provider

**DOI:** 10.1136/bmjresp-2018-000322

**Published:** 2018-10-14

**Authors:** Michela Tinelli, John White, Andrea Manfrin

**Affiliations:** 1 Personal Social Services Research Unit (PSSRU), The London School of Economics and Political Science, London, UK; 2 York Teaching Hospital NHS Foundation Trust, York, UK; 3 Sussex Pharmacy, School of Life Sciences, University of Sussex, Brighton, UK

**Keywords:** quality-adjusted life years, asthma, cost effectiveness, patient reported outcome, community pharmacists

## Abstract

**Introduction:**

A key priority in asthma management is achieving control. The Asthma Control Test (ACT) is a validated tool showing a numerical indicator which has the potential to provide a target to drive management. A novel pharmacist-led intervention recently evaluated and introduced in the Italian setting with a cluster randomised controlled trial (C-RCT) showed effectiveness and cost-effectiveness. This paper evaluates whether the intervention is successful in securing the minimally important difference (MID) in the ACT score and provides better health outcomes and economic savings.

**Methods:**

Clinical data were sourced from 816 adult patients with asthma participating in the C-RCT. The success of the intervention was measured looking at the proportion of patients reaching MID in the ACT score. Different levels of asthma control were grouped according to international guidelines and graded using the *traffic light* rating system. Asthma control levels were linked to economic (National Health Service (NHS) costs) and quality-adjusted life years outcomes using published data.

**Results:**

The median ACT score was 19 (partially controlled) at baseline, and 20 and 21 (controlled) at 3-month and 6-month-follow up, respectively (p<0.01). The percentage of patients reaching MID at 3 and 6 months was 15.8% (129) and 19.9% (162), respectively. The overall annual NHS cost savings per 1000 patients attached to the shift towards the MID target were equal to €346 012 at 3 months and increased to €425 483 at 6 months. Health utility gains were equal to 35.42 and 45.12 years in full health gained, respectively.

**Discussion:**

The pharmacist-led intervention secured the MID in the ACT score and provided better outcomes for both patients and providers.

**Key messages**

- The pharmacist-led intervention secured a minimally important difference (MID) in Asthma Control Test (ACT) score.
- The proportion of patients on target (MID of 3 points) improved at 3-month postintervention and kept improving after 6 months.
- Securing MID in ACT score was linked with cost reduction and quality-adjusted life years increase when moving from not controlled to partially controlled, or from partially to fully controlled.

## Introduction

### The clinical and economic burden of asthma

Recently updated information from the WHO^1^ has confirmed that asthma is still one of the major non-communicable diseases, but that medication can control asthma and appropriate management can enable people with asthma to enjoy a good quality of life. Fink and Rubin^2^ have suggested that management of chronic airways disease is 10% medication and 90% education.

The prevalence of asthma has been increasing since the late 1990s, and it has been estimated that about 400 million people will suffer from asthma by 2025.^3 4^ Asthma accounts for an economic loss of €72 billion annually in the 28 countries of the European Union^5^; this includes the annual costs of healthcare (about €20 billion), the loss of productivity for patients (€14 billion) and a monetised value of disability-adjusted life years loss of €38 billion. In Italy the economic burden of asthma which relates to the degree of control is approximately €5 billion annually. Studies have shown that asthma control has a direct impact on costs^6 7^ and quality of life,^8^ and that there is a clear link between adherence to treatment and asthma control.^9^ The Global Initiative for Asthma (GINA)^10^ suggested that the long-term goals of asthma management are to achieve good symptom control and to minimise future risk of exacerbation, introducing the concept of control-based management, meaning that the treatment is adjusted in a continuous cycle of assessment, treatment and review of patients’ response in terms of control and therefore future risk (attacks and side effects). A key priority is the development of a simple and effective intervention for improving asthma control,^11^ which allows achievement of better socioeconomic and health-related quality of life outcomes.^12 13^

### An effective and cost-effective intervention for patients with asthma: background evidence from a C-RCT

An innovative pharmacist-led intervention for patients with asthma was delivered and evaluated as part of a cluster randomised controlled trial (C-RCT) conducted in 15 of the 20 regions of Italy between September 2014 and July 2015.^14^ It involved 1263 patients with asthma and 283 pharmacists across the territory. The development of the intervention was informed through literature review conducted between 2010 and 2013, which aimed to identify studies reporting pharmacist-led intervention in patients with asthma around the world. The review looked at the following countries: Australia,^15 16^ Belgium,^17^ Canada,^18^ Denmark,^19^ Finland,^20 21^ Germany,^22 23^ Malta,^24^ New Zealand,^25^ Spain,^26^ UK^27 28^ and USA.^29^ The review included randomised and non-randomised studies and allowed identification of the strengths and limitations of each study. Eighteen key points were identified during the review and informed the development of the pharmacist-led intervention, which was informed and retrospectively mapped to the Medical Research Council (MRC) framework^30^ for complex intervention. This intervention represents a bespoke, systematic, structured, face-to-face, pharmacist-led consultation, covering asthma symptoms, medicines used, patients’ attitude towards medicines and self-reported adherence, plus recording of pharmacist-identified pharmaceutical care issues (PCIs).^14^

Community pharmacists were stratified by regions and randomly allocated to group A, who were trained in and delivered the intervention at baseline, and group B, who received training and delivery 3 months later. The procedure was conducted using a computerised random number generation in blocks of 10. Each pharmacist recruited up to five patients, and both groups (A and B) were followed for 9 months. Data were collected at 3-month intervals (at baseline (T0); at 3 months (T3); at 6 months (T6); and at 9 months (T9)).

The primary outcome was asthma control, assessed using the Asthma Control Test (ACT) score (ACT ≥20 represents good control). The secondary outcomes were (1) the number of active ingredients, (2) adherence and (3) cost-effectiveness compared with usual care. Blinding was not possible for either pharmacists or patients. The assessment of outcomes was conducted by a researcher blind to group allocation.

The population overall included more women than men, and the proportion of patients with not-controlled asthma was different between the two groups (A and B), with a median ACT score at baseline of 19 and 18 (see online supplementary material 1). Clinical outcome data (ACT score) were not normally distributed. Mann-Whitney U test was used to assess the ACT score difference between groups before the intervention. Wilcoxon signed-rank test was applied to the pooled sample to assess the ACT score within the group at different time points. The power and sample size calculation was conducted a priori including four different scenarios.^31^

10.1136/bmjresp-2018-000322.supp1 Supplementary data

For cost analysis we used Vervloet *et al* 6 cost data on scheduled healthcare visits to their usual physician and specialist, unscheduled healthcare asthma-related inpatient admissions, emergency visits and emergency contacts with a physician. Healthcare provider costs were estimated from an Italian National Health Service (NHS) perspective. Italian societal costs were sourced from Accordini *et al*.^7^ Health outcome data, in terms of quality-adjusted life year (QALY), a utility measure of disease burden, including both the quality and the quantity of life, were also sourced from the literature looking at published evidence for the Italian population.^8^ The cost for delivering the Italian Medicines Use Review (I-MUR) service per visit per patient was estimated as €40; calculations were based on an average cost of similar services provided in different countries (eg, Canada, Switzerland, UK and USA). All cost data were actualised from 2005 to 2015.

The average annual cost (NHS and society) and utility estimates per patient across groups were calculated looking at the three asthma control categories (not controlled, partially controlled and controlled) as presented in the framework of analysis below (see the Materials and methods section) and linked to the C-RCT data looking at the patient-level ACT scores as reported at three time points (T0, T3 and T6).

The summary cost-effectiveness statistic calculated was the incremental cost-effectiveness ratio (ICER). Uncertainty and variation around the ICER mean were represented by the cost-effectiveness acceptability curve, obtained by resampling the data 1000 times to generate a mean cost and life year or QALY gain from each group, using a non-parametric bootstrap approach. The proportion of resampled data sets for which the calculated ICER lies below a given threshold was interpreted as the probability that the ICER of the intervention is below that threshold.

The key results of the C-RCT showed that the intervention was:

- Effective: The median ACT score was 19 (partially controlled) before the intervention, 20 (controlled) at 3 months after the intervention and 21 at 6 months (p<0.01; see online supplementary material 1). The OR for improved asthma control was 1.76 (95% CI 1.33 to 2.33) in patients who received the intervention versus the ones who did not, and the number needed to treat was 10 (95% CI 6 to 28).
- Cost-effective: The probability of the intervention being more cost-effective than usual care was 100% at 9 months. More details are reported elsewhere.^14^

The intervention also had a positive impact on the following:

- The optimisation of the number of asthma active ingredients used by patients, which was reduced by 8%, and patients’ self-reported adherence to medication, which increased by 40%.

This study had several limitations, among which the evaluation of patients’ adherence was not conducted using a validated tool, but with two questions, for brevity, used also in the English MUR template. The economic evaluation was conducted using secondary data because primary data were not available due to a tight budget.

Although the C-RCT evaluation showed a positive impact of the pharmacist-led intervention on asthma control in terms of improved ACT score (defined as change from not controlled/partially controlled to controlled asthma), the researchers did not measure whether patients who improved their asthma control reported the smallest difference in ACT score that represents a clinically significant change (ie, the minimally important difference, MID) of 3 points in the ACT score. The MID in the ACT score of 3 points is a measure identified by Schatz *et al*,^32^ who suggested that a 3-point difference or change in ACT is clinically meaningful and such a target should be considered when evaluating the clinical performance of asthma interventions.

### Aims and research questions

The focus of the main C-RCT evaluation was to measure the impact of the pharmacist-led intervention on asthma control (in terms of gains in median ACT score) and, following this, possible benefit in terms of cost-effectiveness (see online supplementary material 1 and elsewhere^14^). The purpose of this paper is to take the C-RCT evaluation a step further and measure the impact of the pharmacist-led intervention on MID in asthma control (looking at the proportion of patients reaching a 3-point improvement (MID) in the ACT score). Given the positive outcomes of the C-RCT in terms of both cost-effectiveness (cost per QALYs) and cost savings for the NHS, we also looked at possible benefits of reaching clinical MID, in terms of both health outcomes (QALYs) for the patient and economic savings for the healthcare provider.

The key research questions were the following:

- Is the pharmacist-led intervention provided to patients with asthma effective in securing the MID in the ACT (assessed by a 3-point difference in the ACT score)?
- What is the impact of MID on patients’ quality of life and costs to the healthcare provider?

## Materials and methods

The total C-RCT patient population included in the intention-to-treat analysis was 1263.^14^ A subsample of those (n=816) was used for this analysis (see the C-RCT per-protocol approach).^14^ The proportion of patients on target was compared between groups at baseline and at 3 and 6 months after. Categorical variables were analysed using χ^2^ test for independence.

In the following sections we present the outcome measures and the framework of analysis adopted. Details on the methods uncertainty around cost and QALY parameters are reported above and elsewhere.^14^

### Outcome measures

The primary outcome was the proportion of patients who reached a 3-point improvement (MID) in the ACT score.

The following were the secondary outcomes linked to the MID:

- Health outcome in terms of QALY.
- Costs for the healthcare provider (NHS) to include the cost data on scheduled healthcare visits to their usual physician and specialist, unscheduled healthcare asthma-related inpatient admissions, emergency visits and emergency contacts with a physician. Costs were estimated from an Italian NHS perspective.

### The framework of analysis

**Figure 1:**
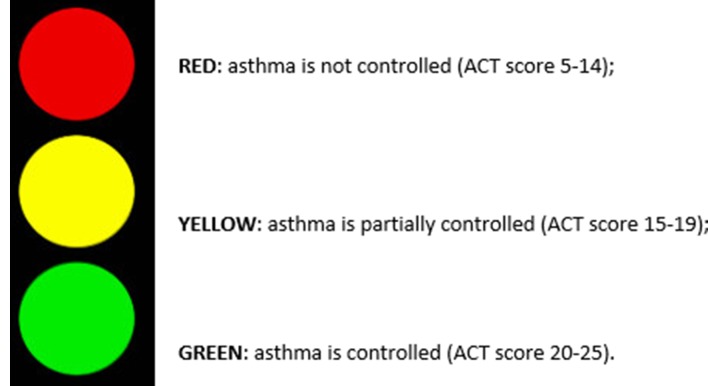
The asthma control groups and their traffic light rating system. The Asthma Control Test (ACT) is a five-item questionnaire that defines three levels of asthma control according to the Global Initiative for Asthma guidelines. Three specific ranges of ACT sources are reported in the figure.^37^ Euroqol-5D, Euroqol 5 dimensions.

**Figure 2:**
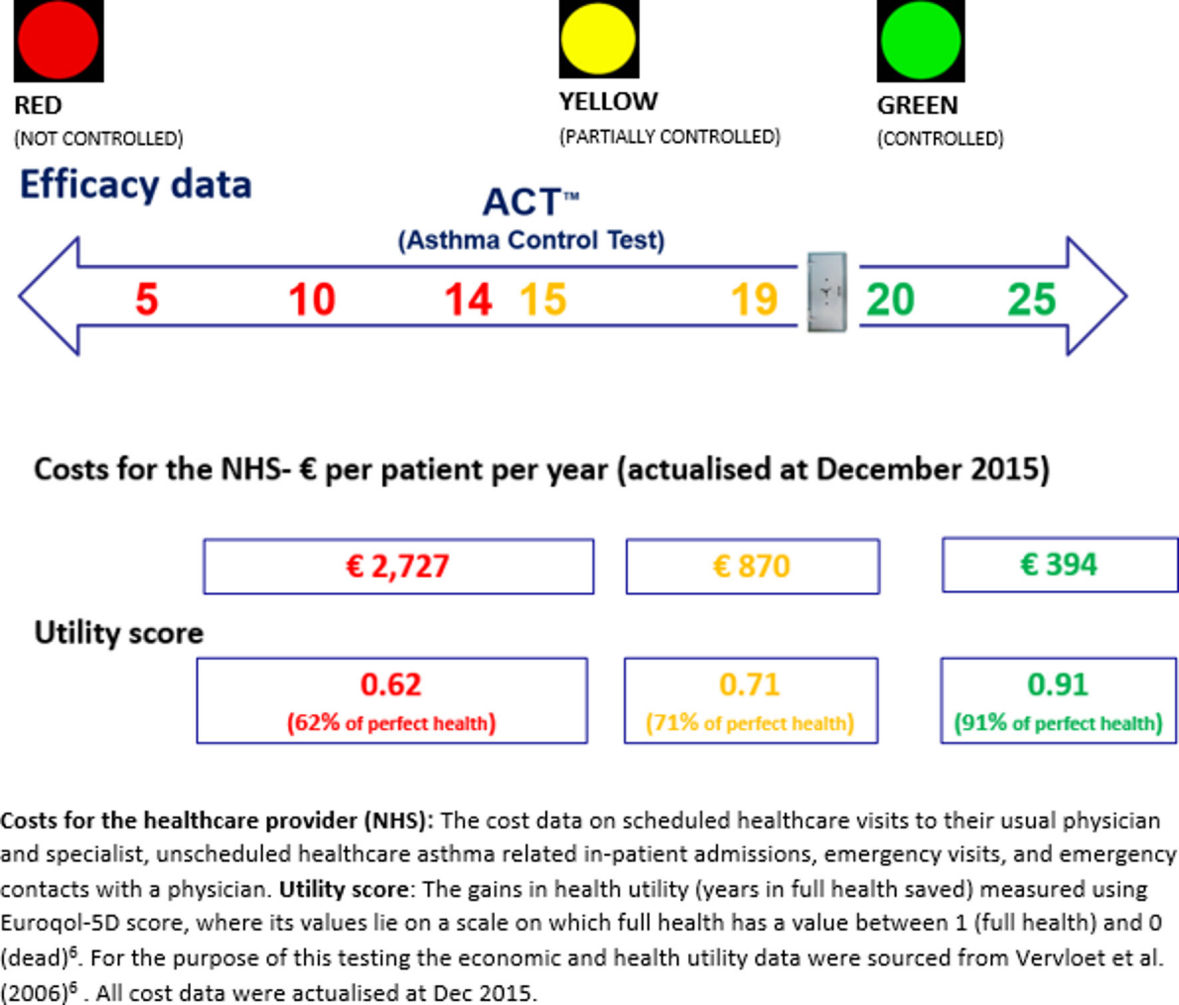
The framework of analysis. Cost for the healthcare provider (National Health Service (NHS)): the cost data on scheduled healthcare visits to their usual physician and specialist, unscheduled healthcare asthma-related inpatient admissions, emergency visits and emergency contacts with a physician. Utility score: the gains in health utility (years in full health saved) measured using the Euroqol-5D score, where its values lie on a scale on which full health has a value between 1 (full health) and 0 (dead).^8^ For the purpose of this testing, the economic data were sourced from Vervloet *et al*.^6^ All cost data were actualised from 2005 to 2015. Euroqol 5D, Euroqol 5 dimensions.

Different levels of asthma control were grouped according to international guidelines^33^ and graded using the *traffic light* rating system (figure 1). Following this, the colour-coded asthma control levels were linked to costs (NHS costs) and health outcome (QALY utility) data using the multidimensional data matrix presented in figure 2.

The average annual cost and utility estimates per patient were extrapolated for not controlled, partially controlled and controlled patients as follows:

- €2727 (95% CI 863 to 4009); €870 (95% CI 306 to 1279); and €394 (95% CI 127 to 579).
- QALY: 0.62 (95% CI 0.60 to 0.64); 0.71 (95% CI 0.70 to 0.72); and 0.91 (95% CI 0.90 to 0.92).

Overall the traffic light rating system allowed the following:

- Colour code-specific stages of asthma control (RED) not controlled (ACT scores 5–14), YELLOW partially controlled (ACT scores 15–19) and GREEN controlled (ACT scores 20–25), defined in terms of clinical, economic and health outcome measures (figure 1).
- Monitor possible changes in asthma control using a typical sequence of traffic light colour phases (eg, the more asthma is under control (moving from RED to YELLOW, and then GREEN) where less money is spent (in terms of annual costs per patient incurred by the NHS) and also more people are able to experience better health) (figure 2).

Specific gains (in costs and health utility) when shifting between different asthma control groups are described in table 1. In order to test whether a gain in ACT score equal to 3 points in ACT score (MID) could have an impact in terms of improved health outcome for the patients (QALY) and cost savings for the healthcare provider (NHS), we considered five different scenarios, described in table 2.

**Table 1:** A shift towards clinical target in asthma control (MID): impact on asthma control, cost savings and gains in health utility (annual estimates per patient)

Possible shifts (current to target scenario)				ACT	Costs for the healthcare provider	Utility score
		Current scenario (ACT)	Target scenario (ACT)	Gain in asthma control (fixed, MID)	Annual saving (2015 euros, per patient)	Gain in health utility (years in full health saved)
1	**RED to RED** 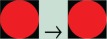	5–10	8–13	3	NA	NA
2	**RED to YELLOW** 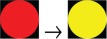	11–14	14–17	3	−1857 (95% CI −2414 to −1393)	0.09 (95% CI 0.088 to 0.092)
3	**YELLOW to YELLOW** 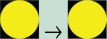	15–16	18–19	3	NA	NA
4	**YELLOW to GREEN** 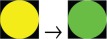	18–19	20–21	3	−2333 (95% CI −3033 to −1750)	0.29 (95% CI 0.28 to 0.30)
5	**GREEN to GREEN** 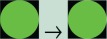	20–21	≥23	3	NA	NA

Possible shifts:a shift in care landscape was considered towards a target scenario where a patient with asthma experienced a clinically significant change in ACT score equal to the MID of 3 points in ACT score.

ACT, Asthma Control Test; MID, minimally important difference; NA (not associated), no change in outcome was captured.

**Table 2:** Possible asthma control cases when securing an MID in asthma control

Possible cases		Description	Possible shifts (current to target scenario)	
			Current scenario (ACT)	Target scenario (ACT)
		*Asthma is not controlled and a gain in ACT score equal to 3 points:*		
1	**RED to RED** 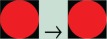	Did not change the asthma control status—still not controlled.	5–10	8–13
2	**RED to YELLOW** 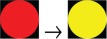	Did change the asthma control status—from not controlled to partially controlled.	11–14	14–17
		*Asthma is partially controlled and a gain in ACT score equal to 3 points:*		
3	**YELLOW to YELLOW** 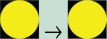	Did not change the asthma control status—still partially controlled.	15–16	18–19
		*Asthma moved from partially controlled to controlled and a gain in ACT score equal to 3 points:*		
4	**YELLOW to GREEN** 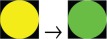	Did change the asthma control status—from partially controlled to controlled.	18–19	20–21
		*Asthma is controlled and a gain in ACT score equal to 3 points:*		
5	**GREEN to GREEN** 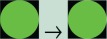	Did not change the asthma control status—always controlled.	20–21	≥23

Possible shifts: a series of shifts in care landscape were considered towards a target scenario where a patient with asthma experienced a clinically significant change in ACT score equal to the MID of 3 points in ACT score.

ACT, Asthma Control Test; MID, minimally important difference.

The ACT scores obtained from the pooled data set (groups A and B before receiving the pharmacist-led intervention)^14 34–36^ were considered for analysis, and the proportion of individuals who met the MID target was calculated after 3-month and 6-month follow-up. A reference population of 1000 patients with asthma was established, and the number of patients on target was calculated for the different shifts. For those who reached the MID target of 3 points, the annual cost saving and utility gains were computed accordingly.

## Results

### Patients demographic and ACT scores

The number of patients included in this analysis was 816 (pooled sample A intervention+B control; see online supplementary material 1). No statistical differences were identified between A and B groups before the intervention. In the pooled sample the median ACT score before the intervention was 19, at 3 months after the intervention 20 (16–23) and at 6 months 21 (17–23). The differences were statistically significant (p<0.01).

### The pharmacist-led intervention provided positive results according to the MID

The proportion of patients who were on MID target and reached the GREEN group at 3 and 6 months moved from 15.8% to 19.9%, respectively. Online supplementary material 2 presents the subsample of people who met a clinically significant change in ACT score equal to the MID of 3 points for the five different scenarios described in table 2.

**Figure 3:**
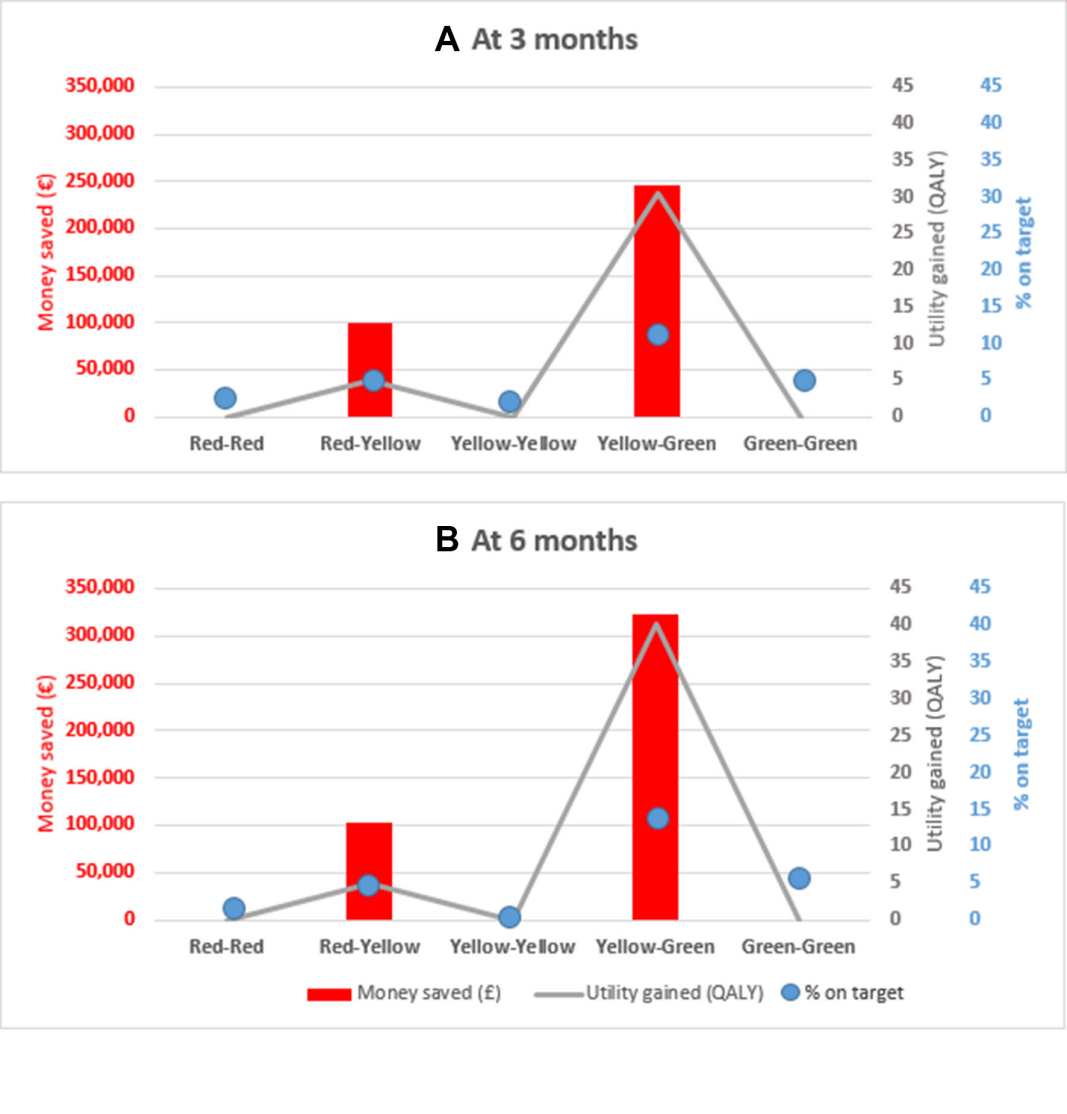
Proportion of patients with asthma on target, annual cost savings and utility gains when securing clinical target in asthma control (minimally important difference) with the pharmacist-led intervention. Population considered=1000 patients with asthma. QALY, quality-adjusted life years. Part A, results at 3 months; Part B, results at 6 months.

When looking at a cohort of 1000 patients with asthma, the overall cost savings attached to the shift towards the MID target (ie, the 349 patients who moved towards better control of their asthma and closed the gaps between RED-YELLOW (150 patients) and YELLOW-GREEN (199 patients)) were equal to €346 012 at 3 months and increased to €425 483 at 6 months. Health utility gains translated into additional 35.42 and 45.12 years in full health gained. Figure 3 presents the annual cost savings and utility gains for the five different cases described in table 2. The changes from RED to YELLOW (shift 2) and YELLOW to GREEN (shift 4) presented the highest benefits in terms of % on target, cost savings and utility gains at both 3 and 6 months (figure 3).

## Discussion

This analysis has demonstrated that the pharmacist-led intervention^14^ can secure an MID in the ACT and in doing so improve patients’ health outcome as well as reduce costs to the NHS. The framework approach to data analysis presented in this paper allows the evaluation of the impact of MID on the ACT score in terms of cost savings for the healthcare provider and gains in health outcome for patients.

### Clinical implications

The introduction of the pharmacist-led intervention and its success in securing a 3-point MID in ACT showed that the economic and utility benefits of better asthma control are evident when shifting from RED-YELLOW and YELLOW-GREEN. Given that the costs associated with controlled asthma are lower than those for managing uncontrolled asthma, any change between not controlled to partially controlled (RED-YELLOW) had greater impact on effectiveness and cost-effectiveness than partially controlled-controlled (YELLOW-GREEN) at 3–6 months of follow-up.

The results showed that the pharmacist-led intervention could promote a shift in current practice towards better asthma control, and this was confirmed by the C-RCT evaluation.^14^ Original C-RCT data showed that the ACT score improved at 3 months after the intervention and went up again at 6 months, suggesting the sustainability of the results.

### Economic implications

The economic evaluation showed that meeting the 3-point MID target can be cost saving for the healthcare provider, and similar results are confirmed in the literature. For example, Schatz *et al* 32 showed that on average a decrease in asthma control of 3 points in the ACT was associated with a subsequent 76% increased risk of excess short-acting β-agonist use and a 33% increased risk of exacerbations with significant impact on the socioeconomic costs. Vervloet *et al* 6 showed that the average cost of asthma management was more than six times higher among patients at the lower levels of the ACT scale with derived ACT score <15 (see figure 2), compared with patients under control, that is, with a derived ACT score of ≥20. A systematic review^13^ looking at the economic burden of asthma suggested that hospitalisation and medication were the most important drivers of healthcare costs, while work loss accounted for the greatest percentage of indirect costs. Moreover, a multinational cross-sectional survey has suggested that the ACT is a predictor of GINA guideline-defined asthma control.^37^ Multiple strategies including, for example, education of patients and doctors, as well as regular follow-up, are required to reduce the economic impact of the disease on both direct and indirect costs. Patients’ education was indicated as the most common PCI identified by pharmacists during the C-RCT project,^14^ where pharmacists followed up their patients for 9 months. As already mentioned Fink and Rubin^2^ suggested that the management of chronic airways disease is 10% medication and 90% education. This analysis of the MID confirmed that even an improvement of 3 points showed an important contribution in clinical, quality of life and economic terms.

### Strengths and limitations of the pharmacist–led intervention and the framework of analysis

The development of the pharmacist-led intervention was informed by the MRC framework. The intervention proved to be an innovative and bespoke service for patients with asthma,^14^ supported by effective and cost-effective outcomes. An additional strength of the paper is the fact that the framework of analysis is based on the National Institute for Health and Care Excellence guidelines and constitutes an easy tool to monitor healthcare performance in terms of clinical, NHS costs and health utility outcomes. The same framework analysis was replicated using a societal perspective (to include loss productivity at work),^7^ and similar cost-effective and cost saving results were confirmed (data are available from the authors on request).

The lack of C-RCT primary data on the economic and health outcome indicators was filled using alternative sources in the literature,^6–8^ still posing limitations in terms of generalisability of estimates, the lack of clarity about either the costing methodology adopted or the utility tariff used to calculate QALY, the components of the cost included (which did not include medication), and the bundle of care considered that may reflect old treatment practices (2004/2005), which are likely to have changed across time. Given the fact that cost and utility estimates were hard to find, a few pragmatic compromises were necessary in modelled approaches. A further limitation of this study is represented by the lack of economic and health outcome data on possible improvement of the MID within specific asthma control categories (eg, 3-point changes that lie within the red area (5–14) did not present any gain in NHS savings and QALYs). Unfortunately the secondary data sources used for this analysis provided aggregate information on the average economic and QALY impacts of asthma control (looking at the three categories not controlled, partially controlled and controlled), whereas these did not allow to have detailed information on the benefits attached to individual ACT scores and estimates for the individual indicators (such as exacerbations, non-elective admissions, visits to the general practitioner and so on). The effectiveness and cost-effectiveness of the pharmacist-led intervention were based on data collected in an experimental setting (C-RCT) with a focus on an adult population in Italy. Another limitation of this study is the potential unmeasured confounding effect that has not been captured in either the clinical or cost data due to the use of the per-protocol analysis approach. More data are needed to monitor the success of the intervention in real-world environment across different healthcare systems and populations. Other limitations of the pharmacist-led intervention have been discussed elsewhere.^14^

## Conclusion

Clinicians and general practitioners looking after their patients with asthma (as well as their professional organisations) can now evaluate the effect and impact of asthma control on clinical, health and costs outcomes, and measure the success of asthma control both in primary and secondary care. Pharmacists could use this evidence-based approach for the provision of a pharmacist-led intervention which appears to be value for money and can align their services with those of other providers as members of the same healthcare professional team. Patients can achieve better asthma control and quality of life, even with a minimum gain of 3-point ACT score. Following discussions of the C-RCT findings with an audience of policy makers, commissioners and practitioners in Italy,^38^ UK^39^ and Europe,^40 41^ it appears that this bespoke pharmacist-led intervention for patients with asthma presents a significant opportunity for success of improving asthma control if implemented in the real-world setting.
