# Supplementary material for: Novel pharmacist-led intervention secures the minimally important difference (MID) in Asthma Control Test (ACT) score: better outcomes for patients and the healthcare provider
Source: BMJ Open Respir Res. 2018 Oct 14;5(1):e000322. doi: 10.1136/bmjresp-2018-000322 (PMC6203066; doi:10.1136/bmjresp-2018-000322)
Supplement: Supplementary data [file bmjresp-2018-000322supp001.pdf]

Supplementary material 1: Patients demographic and ACT scores (per-protocol analysis)

	**Group A**	**Group B**	***p*-value**
Number of patients	400	416	
Female %*	59.8	57.9	0.598
Age range *n* (%)*			0.690
18 to 30	32(8.0)	43(10.3)	
31 to 40	58(14.5)	62(14.9)	
41 to 50	79(19.8)	77(18.5)	
51 to 60	73(18.3)	80(19.2)	
61 to 70	82(20.5)	77(18.5)	
71 to 80	59(14.8)	52(12.5)	
Over 81	17(4.3)	25(6.0)	
ACT scores**			
Median (IQR)	19(15-23)	19(15-23)	0.241

*Chi-square, p<0.05

**Mann-Whitney U test, Median (IQR), p<0.05

Supplementary material 2: Evaluation of success in securing a shift towards clinical target (MID) of asthma control using the pharmacist-led intervention

Possible shifts (current to target scenario)				@ 3 months Total N=1000		@ 6 months Total N=1000		
		Current scenario (ACT)	Target scenario (ACT)	N	% on target	N	% on target	Difference across time (p value)
1	RED to RED	5-10	8-13	26	2.6	16	1.6	0.165
2	RED to YELLOW	15-16	14-17	54	5.4	55	5.5	0.912
3	YELLOW to YELLOW	15-16	18-19	22	2.2	9	0.9	0.026
4	YELLOW to GREEN	18-19	20-21	105	10.5	138	13.8	0.041

**5**	**GREEN to GREEN** →	20-21	≥23	53	5.3	60	6.0	0.519

Possible shifts = A shift in care landscape was considered towards a target scenario where asthma patient experienced a clinically significant change in ACT score equal to the minimally important difference (MID) of 3 points in Asthma Control Test (ACT) score. Note: CI, confidence interval at 95%.
Percentage (%) on target = percentage of people who did the shift AND met a clinically significant change in ACT score equal to the minimally important difference (MID) of 3 points in ACT score. The ACT score obtained from the pooled data set (group A and B before receiving the pharmacist-led intervention) [16-18] was considered for analysis, and the proportion of individuals who met the MID target was calculated after a three- and six-month follow-up. A reference population of 1,000 asthma patients was considered and the number of patients on target was calculated for the different shifts.
